# Selecting and tailoring implementation interventions: a concept mapping approach

**DOI:** 10.1186/s12913-020-05270-x

**Published:** 2020-05-06

**Authors:** Elaine Yuen Ling Kwok, Sheila T. F. Moodie, Barbara Jane Cunningham, Janis E. Oram Cardy

**Affiliations:** 1 School of Communication Sciences and Disorders, University of Western Ontario, Elborn College, 1201 Western Road, London, Ontario N6G 1H1 Canada; 2 School of Communication Sciences and Disorders, Western University and The National Centre for Audiology, Western University, Elborn College, RM 2262K, 1201 Western Road, London, Ontario N6G 1H1 Canada

**Keywords:** Implementation intervention, Speech-language pathologist, Concept mapping, Practice-based research, Outcome measurement, Population outcomes; health outcomes; stakeholder engagement, Implementation planning

## Abstract

**Background:**

To improve the uptake of research into practice, knowledge translation frameworks recommend tailoring implementation strategies to address practice barriers. This study reports our experience pairing the Theoretical Domains Framework with information from multiple stakeholder groups to co-develop practice-informed strategies for improving the implementation of an evidence-based outcome measurement tool across a large community health system for preschoolers with communication impairments.

**Methods:**

Concept mapping was used to identify strategies for improving implementation of the Focus on the Outcomes of Communication Under Six (FOCUS) in Ontario Canada’s Preschool Speech and Language Program. This work was done in five stages. First, we interviewed 37 speech-language pathologists (clinicians) who identified 90 unique strategies to resolve practice barriers to FOCUS implementation. Second, clinicians (*n* = 34), policy-makers (*n* = 3), and members of the FOCUS research team (*n* = 6) sorted and rated the strategies by importance and feasibility. Third, stakeholders’ sorting data were analyzed to generate a two-dimensional concept map. Based on the rating data from stakeholders, we prioritized a list of strategies that were rated as highly important and highly feasible, and summarized the practice barriers addressed by each of the prioritized strategies. Fourth, we validated these findings with stakeholders via an online survey. Fifth, the mechanisms of action of the prioritized list of strategies were considered based on available evidence from the Theoretical Domains Framework and associated behavior change literature.

**Results:**

Stakeholders categorized the 90 unique implementation strategies into a six-cluster concept map. Based on stakeholders’ ratings, a list of 14 implementation strategies were prioritized. These implementation strategies were reported to resolve barriers within the *environmental context and resources* and *beliefs about consequences* domains of the Theoretical Domains Framework. All but one of the prioritized strategies have a demonstrated link in resolving existing barriers according to the behavioral change literature.

**Conclusions:**

Our study contributes to a growing literature that demonstrates the process of tailoring implementation strategies to specific barriers. Practical drawbacks and benefits of using concept mapping as a way to engage stakeholders in implementation research are discussed.

## Background

The knowledge-to-action framework [1] is a widely adopted framework to support the implementation of best evidence into practice. This framework offers a step-by-step approach to improving the uptake of evidence into practice. Once barriers to uptake are identified, implementation strategies are selected and tailored to address them [1]. Implementation strategies are methods (or the “how to”) for promoting the use of research evidence in practice [2]. The literature offers as many as 73 implementation strategies that vary in their impact and feasibility [3, 4], and there are different methods researchers can take to select appropriate strategies.

One way to select implementation strategies is to consult the research literature and apply explicit theories [5]. Once barriers are identified, appropriate theories can be used to guide the design of implementation strategies that will address the barriers and lead to practice change (e.g. to target a lack of self-efficacy, Social Cognitive Theory suggests strategies such as peer modelling) [6]. A major benefit of this approach is that theory can be used to predict and explain the mechanism by which implementation strategies will impact barriers, and therefore, may increase the likelihood of changing behaviour [5, 7]. Frameworks that summarize behavioral change theories have been developed to help support researchers in this process. Of note, the Theoretical Domains Framework (TDF) consolidated 33 psychological theories [8] to offer a theory-driven way of characterizing implementation barriers and facilitators [9]. The TDF describes 14 unique domains of factors that impact the implementation of evidence-based practices (e.g. knowledge, skills, emotion) [9, 10]. Emerging work has expanded the use of the TDF beyond the description of these factors. For example, the TDF domains have been linked to specific behavior change techniques [11, 12], which are described as the components (or the “active ingredients”) that constitute behavior change interventions [13]. Furthermore, through an expert consensus approach, the mechanisms of action of the behavior change techniques have been identified [14]. These mechanisms of action describe how (i.e., the process by which) different behavior change techniques can resolve implementation barriers [14].

Selecting implementation strategies based on theoretical frameworks, such as the TDF and behavior change theories, has limitations. One is that the conceptual link between the domains on the TDF and behavioral change techniques is still emerging. To date, not all TDF domains have been linked with specific behavior change techniques [11]. In other words, the literature may not offer guidance on the appropriate implementation strategies for some barriers (e.g., skills, social/professional identity). More importantly, behavioral theories that apply in controlled experimental settings may be difficult to translate into real-world implementation strategies where naturally occurring practical or contextual constraints are present and cannot be modified [15, 16].

Another way to select implementation strategies is to collect data related to stakeholders’ experiences and preferences [16]. Using this type of approach, stakeholders are engaged in the process of identifying implementation barriers and strategies to address them from the beginning of the research process. Including stakeholders in the process “up front” has been shown to positively impact implementation and clinical outcomes, perhaps because specific practice contexts and barriers within them are considered [17–19]. Engaging stakeholders in selecting implementation interventions is also beneficial because they are the intended knowledge-users. When stakeholders’ experiences and opinions are integrated into decision-making processes, the selected implementation intervention strategies may be more important to knowledge-users and more feasible at their organizational context [16, 18].

Concept mapping has been proposed as one potential approach for engaging stakeholders in the design of implementation strategies [20]. In concept mapping, stakeholders participate in brainstorming, sorting, and rating activities to reach a consensus on the best strategies to improve implementation [20, 21]. The concept mapping approach has several benefits: (i) it offers clear and structured activities for data collection; (ii) these activities encourage equal participation from all stakeholders; (iii) the collected data allow for the identification of group consensus; and (iv) the analyses are flexible and allow for balancing the opinions from multiple stakeholder groups [21]. How the concept mapping approach may be applied for tailoring implementation strategies is currently not clear.

To be effective, implementation strategies should be selected based on practice barriers and theories of implementation, and should be tailored to the contexts in which they will be implemented [1, 16, 20, 22]. The purpose of this study was to illustrate a research approach that considers both research evidence (i.e., the TDF) and stakeholder perspectives and feedback to identify strategies to improve implementation of a new outcome measurement tool across a large preschool speech-language health system. We asked two specific questions: (i) how can stakeholders be engaged to identify barrier-specific implementation strategies and (ii) is there evidence to suggest the implementation interventions generated by stakeholders will resolve practice barriers? This study will illustrate how the concept mapping approach may be applied to answer these research questions. The discussion highlights the necessary modifications, benefits, and practical limitations to be considered when applying the concept mapping methodology.

## Methods

### Study setting

In Ontario, Canada, a provincial outcome monitoring protocol was implemented by the Ontario Preschool Speech and Language (PSL) Program. This program serves over 60,000 children annually across 30 service regions. Since 2012, speech-language pathologists (clinicians) have been required to collect parent-report outcome data using the *Focus on the Outcomes of Communication Under Six* (FOCUS) at 6 months intervals for all children 18 months of age and older. The FOCUS is a tool designed to measure changes in communicative participation skills for preschool children receiving speech and/or language therapy [23].

The FOCUS was developed and validated by engaging knowledge users (i.e. clinicians and parents of preschoolers with speech and language impairments) throughout the development process [24]. As a measurement tool, the FOCUS has good internal consistency, reliability, and validity (construct, convergent, and discriminant) [24, 25] and its items reflect the Activity and Participation components of the World Health Organization’s (WHO) International Classification of Functioning, Disability and Health (ICF) framework [24]. As a criterion-referenced measurement tool, the FOCUS allows clinicians to measure change within an individual child by providing validated reference values that indicate whether a child made clinically meaningful change during an intervention period [23]. In 2015, based on the feedback from clinicians working in the PSL Program, the FOCUS was shortened from 50 to 34 items [26].

Despite its strong psychometric properties and initial implementation efforts, the adoption and consistency of use of the FOCUS continued to vary across the 30 PSL Program regions [27, 28]. For instance, clinicians at some PSL program regions stopped collecting and reporting FOCUS data. In 2018, we began working to understand the contextual challenges related to implementation of the FOCUS, and to identify ways to improve implementation. In our first study, we interviewed 37 clinicians representing the 30 PSL Program regions to learn their perceived facilitators and barriers for implementing the FOCUS (Manuscript under review). Clinicians reported major barriers in three TDF domains: environmental context and resources, beliefs about consequence, and social influences. In the present study, we used concept mapping to select implementation strategies to target the barriers identified by the clinicians.

### Participant recruitment

We identified three stakeholder groups involved in the implementation of the FOCUS in the Ontario PSL Program. Stakeholders included clinicians (knowledge users), representatives from the PSL Program (policy makers and managers), and the FOCUS research team, whom were responsible for developing, validating, and initial implementation of the FOCUS. Purposeful sampling was used to recruit clinicians. We contacted the clinical coordinators (similar to regional managers) from the 30 PSL Program regions. These coordinators forwarded recruitment emails to speech-language pathologists who worked within their respective regions. Clinicians were asked to contact us by email if they were interested in participating. Using this method, we were contacted by 37 clinicians, all of whom agreed to participate in telephone interviews. The sample included at least one clinician from each of the 30 regions, providing representation from across the PSL Program. At the time of the study, there were 400 speech-language pathologists working in the PSL Program, which means our sample represented 9.25% of potential participants. We cannot report response rates as there was no way for us to verify whether all clinicians received the email invitation to participate. Convenience sampling was used to recruit policy makers (*n* = 3) and members of the FOCUS research team (*n* = 6).

### Procedure

Concept mapping provides a rigorous approach that engages stakeholders in a series of sequential tasks. It is fundamentally a mixed-methods approach that involves multiple sequential stages. These include: (1) brainstorming and statement analysis, (2) structuring of statements (sorting and rating) by stakeholders, (3) concept mapping analysis, and (4) data interpretation [21, 29, 30]. Qualitative steps include brainstorming and sorting, quantitative steps include the multidimensional scaling, cluster analysis, and computation of a concept map (see Additional file 1 for our reporting guideline checklist [31]).

#### Stage 1: brainstorming and statement analysis

The goal of this stage was to generate a list of strategies that would improve implementation of the FOCUS based on stakeholders’ experiences and perspectives. Over telephone interviews, 37 clinicians brainstormed strategies to improve the implementation of the FOCUS using the prompt “*One specific thing that will help me complete and submit the FOCUS regularly is …*.” In addition, clinicians were asked to elaborate on the barrier(s) that their strategies would address. This stage was completed via telephone interviews to facilitate participation across a wide geographic region. Phone interviews were recorded and transcribed verbatim, but pseudonyms were used for identifying information. A research assistant reviewed all transcripts to ensure transcription fidelity.

#### Stage 2: structuring the statements

Data were collected from stakeholders to develop a common framework for conceptualizing and prioritizing the suggested implementation strategies. We invited clinicians (*n* = 37 who participated in the brainstorming stage), policy-makers (*n* = 3 representatives from the PSL program), and members of the FOCUS research team (*n* = 6) to sort and rate the 90 implementation strategies over the web-based Concept System Global Max™ software [32].

For the sorting task, participants were instructed to sort the strategy statements into categories that made sense to them and to generate a label for each category they created. Participants were instructed not to create a miscellaneous category nor to sort strategies by degree of importance or feasibility. There was no limit to the number of categories participants could create, but we suggested that most complex ideas could be summarized within 20 categories.

For the rating task, clinicians were asked to rate the importance of each strategy statement on a scale ranging from 0 (not important at all) to 5 (extremely important) based on the impact each strategy would have on the implementation of the FOCUS. As well, all participants (clinicians, researchers, and policy makers) were then asked to rate each strategy statement on its feasibility using the scale 0 (not feasible at all) to 5 (extremely feasible). Clinicians were asked to consider the feasibility of implementing the strategies within their practice environments whereas policy makers and FOCUS research team members were asked to consider the feasibility of adopting/implementing the strategies from their administrative and research perspectives.

#### Stage 3: concept mapping analysis

Based on how participants sorted and rated the 90 suggested implementation strategies, we generated a conceptual framework and prioritized the list of strategies. To create a concept map, sorting data from all participants was entered into CS Global MAX™ software (Concept System Inc., Ithaca, NY) to create a similarity matrix. In this matrix, a numerical value of similarity was assigned to any two strategy statements based on the number of participants who sorted them into the same category. Through multidimensional scaling, the value of similarity between any two statements was converted into distance (expressed as *X,Y* coordinates) on a two-dimensional concept map (the higher the similarity value, the shorter the distance between the statements). The *X,Y* coordinates of every statement were then analyzed using hierarchical cluster analysis, which grouped statements located closer together into the same category. In other words, statements that were grouped together more frequently by the participants appeared closer on the concept map and had a higher likelihood of being included in the same category during the cluster analysis, whereas statements that were less frequently grouped together appeared further from each other on the concept map, and had a lower likelihood of being included in the same category [21].

The next step was to determine the most appropriate number of categories to include in the concept map. To this end, we first reviewed participants’ sorting data to determine whether there was a consensus on the number of categories created by each participant. The most common number of categories created by participants was seven (*n* = 14 of our participants created seven categories). To determine whether there was a different number of categories that better represented the data, we also created concept maps that included 4–10 categories (using 7 ± 3, the interquartile range of our sample). These maps were reviewed by the authors starting with the map that had 10 categories and moving to the map that had four. Each time the number of categories was reduced, we reviewed the contents of the new categories to determine whether the statements were conceptually related.

To prioritize the implementation strategies, we created *Pattern Match* and *Go-Zone* graphs using the CS Global MAX™ software. The Pattern Match graphs are ladder graphs that illustrate the correlation between two sets of ratings. In our case, we explored: 1) the correlation between clinicians’ ratings of importance versus feasibility, and 2) the correlations between clinicians’ rating of importance versus policy makers and researchers’ ratings of feasibility. The former was explored to ensure strategies that were important to clinicians were perceived as feasible in clinical settings. The latter was explored to see if strategies that were important to clinicians were feasible from the perspectives of policy makers and researchers (i.e. by those making decisions about policy and resource allocation and those supporting research and implementation). These Pattern Match graphs allowed us to visualize data at a category level. The rating plotted on each side of the Pattern Match graph was generated by averaging the ratings of all strategies within a category. To present the importance and feasibility of each strategy, *Go-zone* graphs were plotted. *Go-zone* graphs present each strategy by plotting the feasibility rating from policy makers and researchers (y-axis) against the clinicians’ ratings of importance (x-axis). This means strategies that were highly feasible and important appear in the top-right quadrant.

#### Stage 4: data interpretation

To create labels for the categories identified in the concept map, the authors reviewed strategies within each category and considered the labels suggested by our participants. We also considered strategies within each category that contributed most to the uniqueness of that category (i.e. statements that were heavily loaded onto one category and contributed less to other categories). After determining the label for each category, a brief description was written to summarize the strategies within each category. As a member-check step, stakeholders reviewed and approved of these labels and descriptions in an online survey (see Additional file 2).

To determine a list of implementation strategies that were rated as both feasible and important by stakeholders, we first reviewed the Pattern Match graphs to identify the categories on the concept map that all stakeholders agreed to be important and feasible. We then consulted the Go-zone graphs of these categories and identified strategies that were rated highly on both importance and feasibility (i.e. those that were in the top-right quadrant of the graph). Lastly, we reviewed importance and feasibility ratings for each suggested strategy to identify those that received high ratings (> 4 points) from all stakeholder groups. These selected strategies were further prioritized based on the importance and feasibility ratings.

We added the following steps to the traditional concept mapping methodology in order to understand the barriers being addressed by the implementation strategies. In our interviews (described in Stage 1 above), clinicians were asked to report what specific barrier would be addressed by each implementation strategy they generated. In this phase, we reviewed all interview transcripts to identify clinicians who recommended the implementation strategies on the prioritized list. We then reviewed those interview transcripts and selected representative quotes to illustrate the barriers clinicians reported. Through discussions, the authors reached consensus on the specific TDF domain associated with the described barrier.

Additionally, we validated the concept map and the prioritized list of implementation strategies (along with the reported barriers) with our stakeholders who participated in the previous steps in this project. Using an online survey (see Additional file 2), stakeholders rated their level of agreement with our findings on a Likert scale from 0 = strongly disagree to 5 = strongly agree. Participants were given an opportunity to provide written feedback to our findings in open-ended questions. An a priori threshold of consensus was defined to be 85% agreement amongst survey respondents, which is considered to be a more conservative threshold compared to published Delphi studies [33].

#### Stage 5: Verify the mechanisms of action of the prioritized list of implementation strategies

The final step in our approach aimed to verify that strategies considered to be important and feasible by stakeholders were also appropriate from a theoretical perspective (i.e., had evidence demonstrating they could be used to resolve the implementation barriers). The research team first mapped the prioritized implementation strategies in this study to the behavior change techniques, which are published behavioral change intervention methods [13]. We then reviewed the mechanisms of action associated with each implementation intervention strategy. Mechanism of action is defined as “the processes through which behavior change occurs” [14]. We considered whether the prioritized implementation intervention strategies had a mechanism of action known to impact the purported implementation barriers. Implementation intervention strategies prioritized by stakeholders that did not have empirical evidence to suggest potential for impact on the purported barriers were removed. Intervention strategies that were supported by the literature were retained as recommended strategies.

## Results

**Fig. 1:**
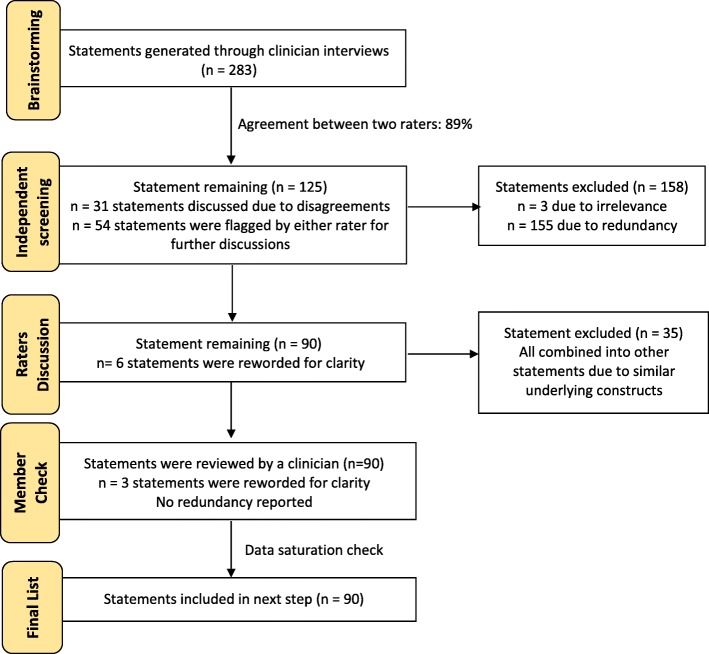
Strategy statements preparation workflow

Thirty-seven clinicians brainstormed strategies for improving implementation of the FOCUS in the PSL Program (years of experience, median = 9; range 1–24). Clinicians generated 282 strategy statements to improve implementation. The following steps were taken to prepare the strategy statements for the sorting and rating stage (also illustrated in Fig. 1):

1. To determine relevance and redundancy, strategy statements were independently reviewed by the first and third author who had experience in clinical settings where the FOCUS use was mandated.
2. Both raters agreed to exclude 158 strategy statements due to redundancy or irrelevance but disagreed on the eligibility of 31 statements (interrater agreement = 89%, Kappa = 0.78). Additionally, 54 statements were identified by either rater as needing further discussion.
3. After discussion, both raters agreed to exclude an additional of 35 statements due to redundancy and to modify six statements to improve clarity (*n* = 90 strategies were included).
4. As a member-check step, the included strategy statements were sent to a clinician in the PSL program who verified that there was no redundancy, but suggested editorial changes to 3 statements to improve clarity.
5. A final list of 90 clear and unique strategy statements was entered into the web-based Concept System Global Max™ software [32]. As the main goal of this stage was to generate a list of ideas “that represent the diversity of thought” [21], we reviewed our interview transcripts to verify that item saturation was reached. This was indeed the case, as our final four interviews did not generate any new strategies.

**Fig. 2:**
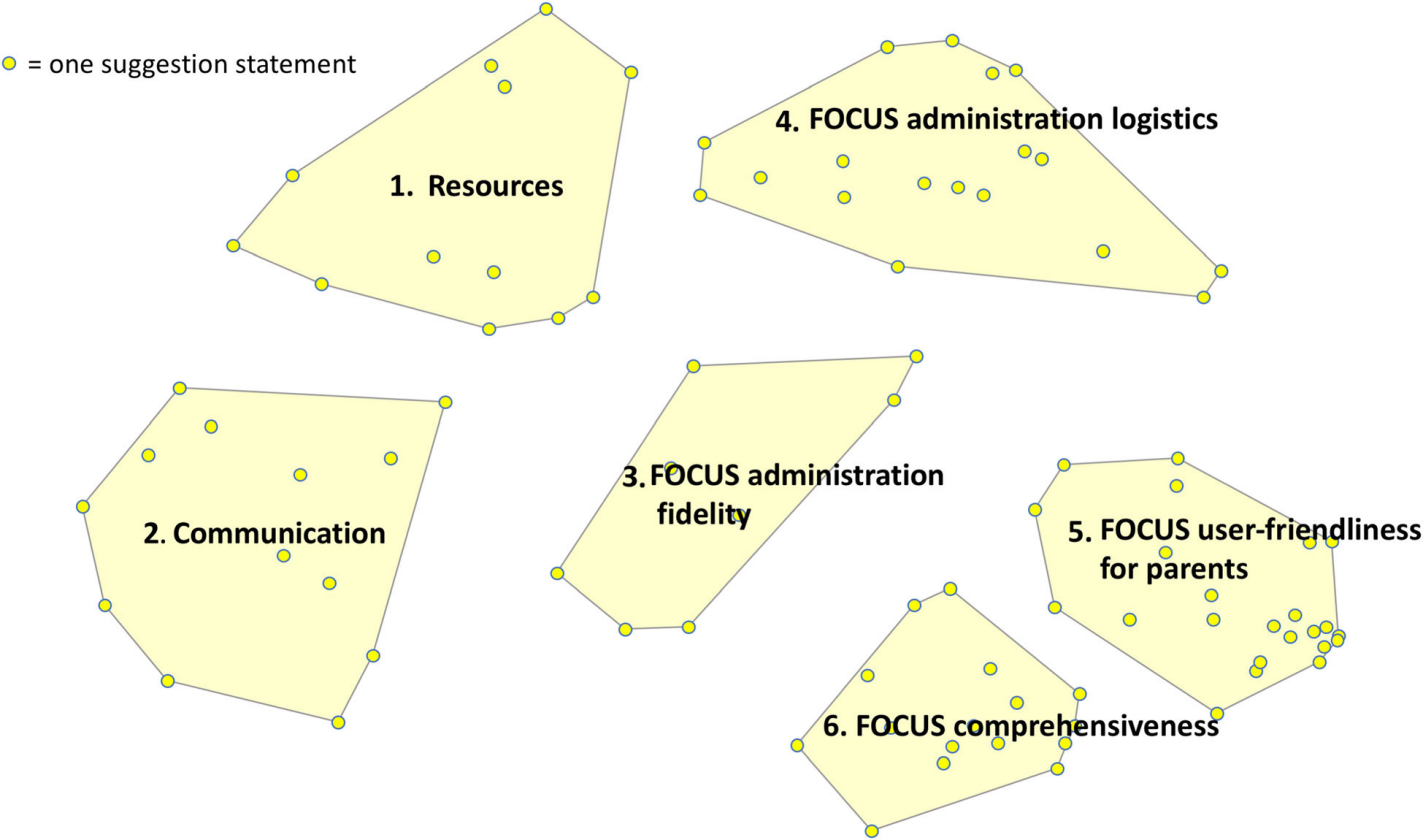
Concept map of the 90 implementation strategies summarized into 6 categories

Based on participants’ sorting data, the list of 90 unique implementation strategies was best represented in 6 categories (see Fig. 2 and Table 1; Additional file 3 provides the full list of strategies within each category), including:

1. Resources: provide additional financial supports and personnel support
2. Communication: share information with frontline staff and maintain ongoing communication between the Program and clinicians
3. FOCUS administration fidelity: improve the consistency with which the FOCUS is introduced to parents, scored, interpreted, and used to support clinical practice
4. FOCUS administration logistics: facilitate the process of FOCUS data collection as well as the administrative schedule of the FOCUS
5. FOCUS user-friendliness for parents: improve clarity, readability, and literacy level of the FOCUS so it is easier for parents to complete
6. FOCUS comprehensiveness: ensure the FOCUS is applicable and appropriate for all children and families

**Table 1:** Example strategies for each of the 6 categories on the concept map

**1. Resources** Hire more clinicians Provide more funding for clerical support for data entry	
**2. Communication** Share what is done at a program level to evaluate program effectiveness using the FOCUS Share information on how other agencies/clinicians are using FOCUS data clinically	
**3. FOCUS administration fidelity** Create a poster/visual display that explains the purpose of the FOCUS Make sure FOCUS scores can support functional/clinical activities	
**4. FOCUS administrative logistics** Offer an electronic fillable FOCUS form (e.g. on tablet/iPad/online/laptop) Re-examine the frequency and timing with which the FOCUS should be completed	
**5. FOCUS user-friendliness for parents** Improve readability of the FOCUS (e.g. increase the font size and bubble size, shading of items) Simplify the wording of FOCUS items so they are appropriate for all reading levels	
**6. FOCUS comprehensiveness** Make sure FOCUS items apply to children at all levels of communicative function Have separate sections for items that ask about verbal vs non-verbal forms of communication	

Six clinicians did not accept our invitation to complete the online sorting and rating tasks, so we recruited three additional clinicians in the PSL program through personal connections (*n* = 34 completed the online tasks). All invited policy makers and FOCUS research team members completed the online tasks. Despite our instructions and reminders, seven participants (*n* = 4 clinicians, *n* = 3 policy makers) sorted the strategy statements into importance/feasibility categories (e.g. by creating categories such as “Not feasible” or “Not important”) and their data were excluded from concept map analysis. All participants rated strategies on importance or feasibility (*n* = 43).

**Fig. 3:**
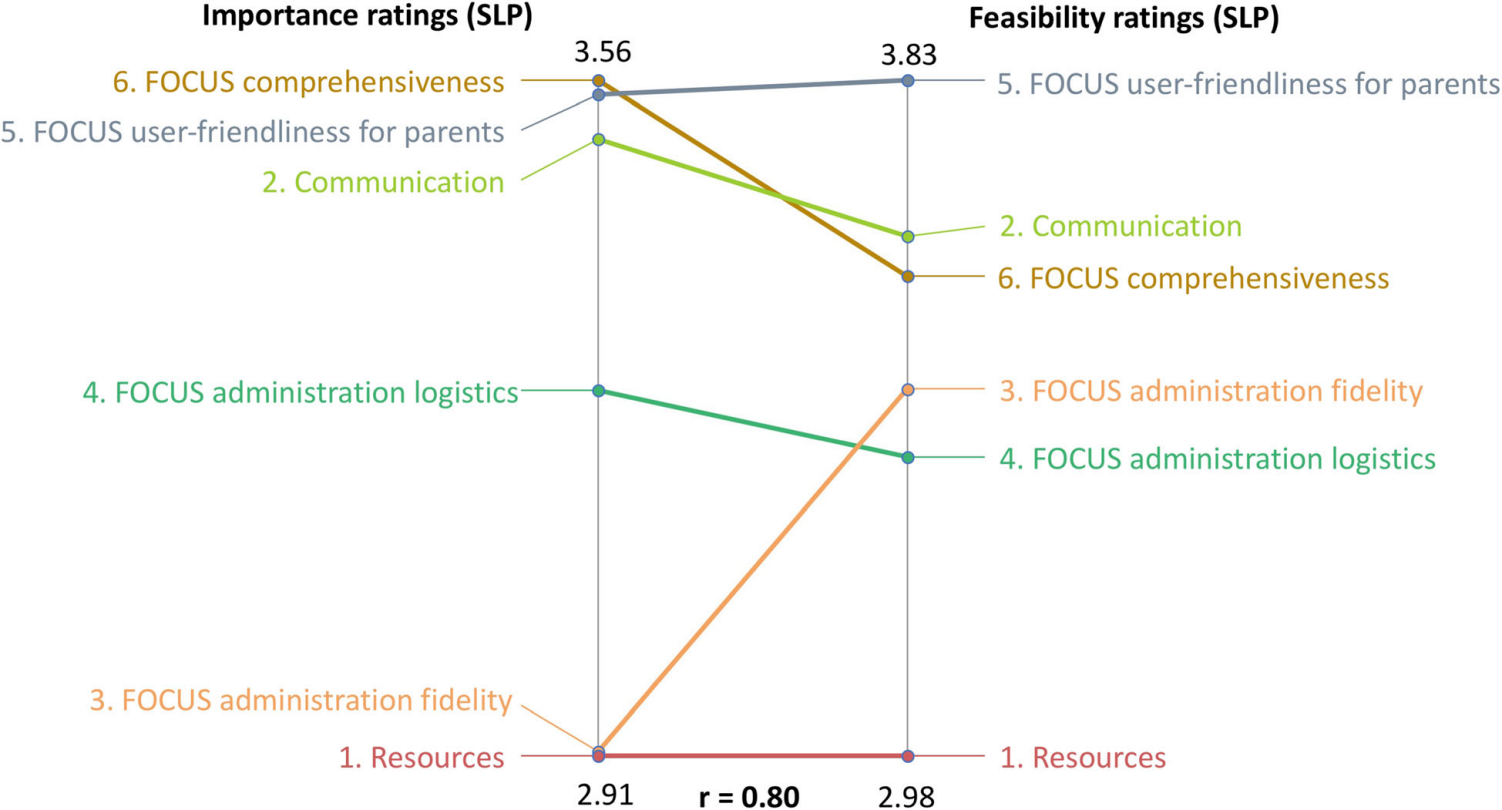
Pattern Match graph of clinicians’ ratings on importance (right) versus feasibility (left)

Clinicians’ ratings for importance and feasibility were highly correlated across categories, *r* = 0.80 (see Fig. 3). For most categories, the importance (right) and feasibility (left) ratings were similar. One category, *FOCUS Administration Fidelity*, was the exception. Clinicians rated this category as feasible but not important for implementing the FOCUS.

**Fig. 4:**
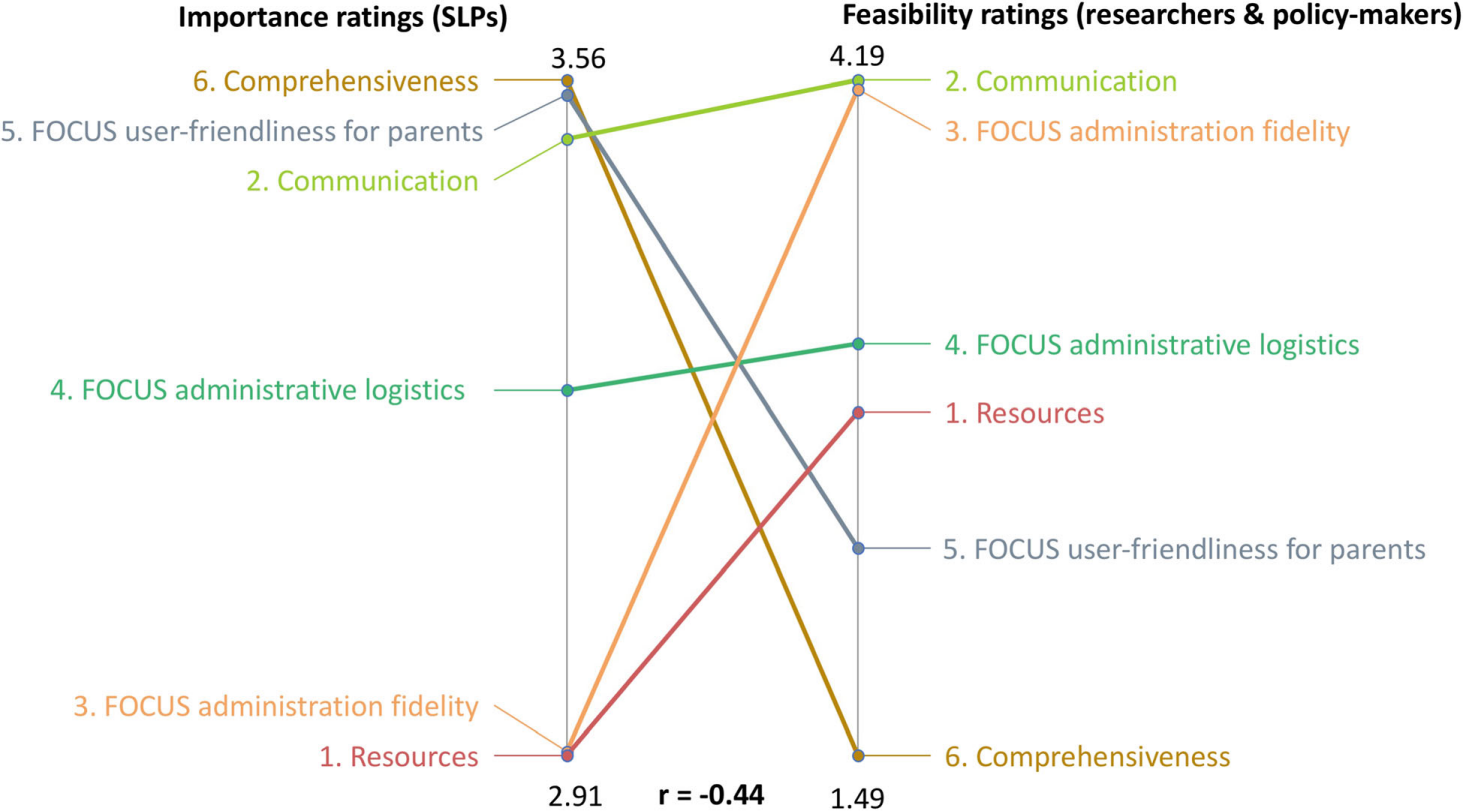
Pattern Match graph of clinicians’ importance ratings (right) versus policymakers and researcher’s feasibility ratings (left)

In contrast, there was a moderate negative correlation between clinicians’ importance ratings and feasibility ratings from both policy makers and researchers, *r* = − 0.44 (see Fig. 4). This means that some categories rated as most important by clinicians (i.e. *FOCUS comprehensiveness* and *FOCUS user-friendliness for parents*) were rated as least feasible by policy makers and researchers. The category *Communication* was rated as highly important and feasible by all stakeholder groups and *FOCUS Administration Logistics* were rated as fairly important and feasible by all groups.

**Fig. 5:**
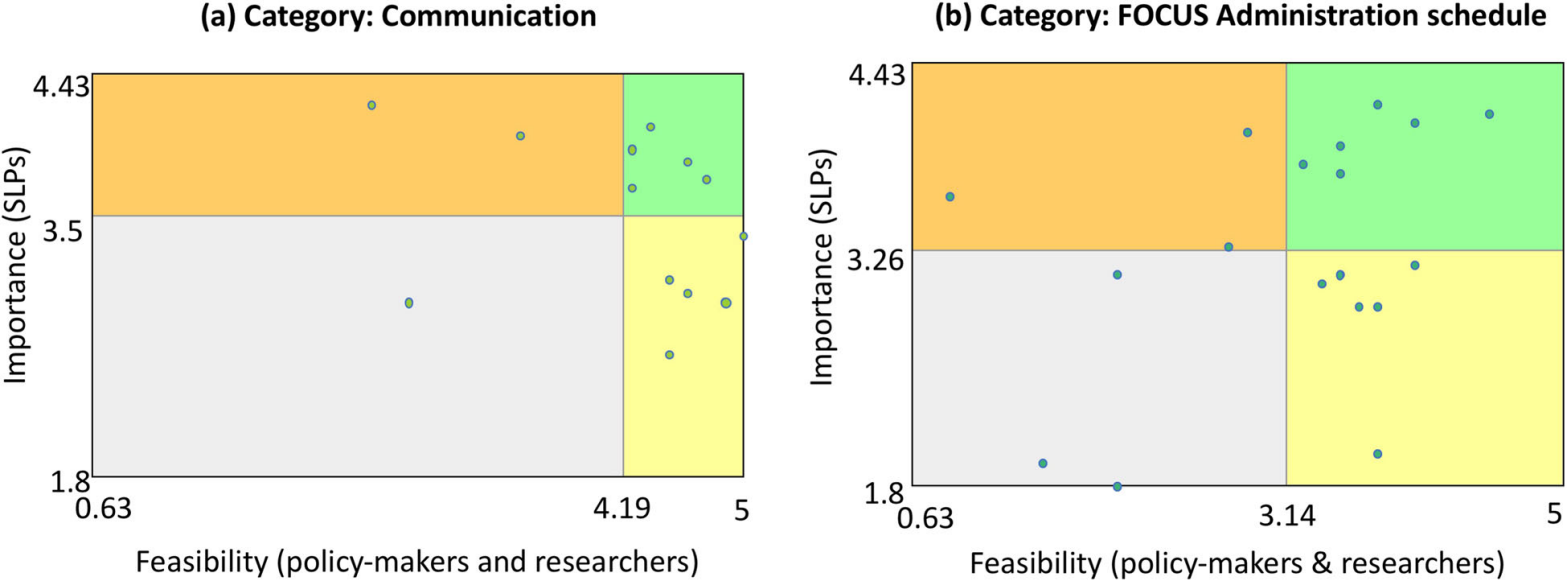
Go-zone display of the (**a**) Communication and (**b**) FOCUS Administration schedule categories. Each dot represents one strategy

Given that two categories (*Communication* and *FOCUS Administration Logistics)* were rated highly on importance and feasibility by all stakeholder groups, we created *Go-zone* figures for strategies in these two categories (Fig. 5a & b). Five strategies in the *Communication* category and six in the *FOCUS Administration Logistics* category fell into the top right quadrant of the *Go-zone* figures. To ensure that we did not leave out strategies that were important and feasible in other categories, we also reviewed clinicians’ ratings of importance and policy makers’ and researchers’ ratings of the feasibility for all other strategies. A cut score of four points (out of five) was used as a conservative estimate of importance/feasibility. Three additional strategies were identified using this approach. Prioritized strategies are presented in Table 2.

**Table 2:** Strategies rated as both important and feasible by all stakeholder groups

Priority	Strategies	Importance	Feasibility	Reported Benefits
1	Offer an electronic fillable FOCUS form (e.g. on tablet/iPad/online/laptop)	4.1	4.5	Improves data collection/submission environment
2	Share what is done at the ministry level to look at program effectiveness using the FOCUS	4.1	4.4	SLPs will know what happens to the FOCUS data they collect and submit
3	Make translations of FOCUS available	4.1	4.4	The data collected from FOCUS will be clinically valid
4	Improve readability of the FOCUS (e.g. increase the font size and bubble size, shading the items)	4.1	4.3	Improves data collection/submission environment
5	Make sure FOCUS scores can support functional/clinically-related activities (e.g. helping clinicians form goals)	4.1	4.1	SLPs will know how they can use the FOCUS data in their practice
6	Offer a way for FOCUS to be completed and submitted by parents at home e.g. online/over the phone	4.1	4	Improves data collection/submission environment
7	Keep the dialogue open with SLPs to see what can be improved/ changed	4	4.3	Research on the FOCUS will incorporate clinical expertise, and be more relevant to practice
8	Provide a way that automatically calculates scores/statistics of FOCUS (including change scores from the last FOCUS and the subscale scores)	4.2	3.8	Improves data collection/submission environment
9	Make sure FOCUS is valid even if different parents/caregivers/SLPs are completing them	3.9	4.6	The data collected from FOCUS will be clinically valid
10	Create an electronic system that streamlines all administration of FOCUS (e.g. can see all FOCUS of the same child in tabs)	3.9	3.5	Improves data collection/submission environment
11	Share successful research findings with the use of FOCUS (specify the details of the intervention and how FOCUS data was collected)	3.8	4.8	SLPs will know how submitted FOCUS data was used in clinical research
12	Change the schedule of FOUCS such that administration is timed to clinical appointments (e.g. assessment/intervention/discharge) rather than saying every 6 months	3.8	3.3	Improves data collection/submission environment
13	Remove the need to transfer FOCUS score by having an app that connects FOCUS data to the ministry (i.e. remove the need to transfer paper to electronic format)	3.8	3.5	Improves data collection/submission environment
14	Provide more timely feedback about FOCUS outcomes to SLPs (rather than at PSL meetings only)	3.7	4.3	SLPs will know what happens to the FOCUS data they collect and submit

Clinicians were asked during the telephone interviews to elaborate on the barriers their implementation suggestions would address. Based on clinicians’ reports, we matched the barriers addressed by each of the 14 strategy statements to the TDF domains (see the Reported Benefits column in Table 2; Additional file 4 provides example quotes from the interviews). The selected strategies addressed two TDF domains, namely *beliefs about consequence* (*n* = 7) and *environmental context and resources* (*n* = 7). The seven strategies reported to address clinicians’ *beliefs about consequence* included sharing information on the collected FOCUS data and making sure the FOCUS provides clinically relevant information. The remaining seven strategies related to *environmental context and resources* focused on improving and digitizing the process of FOCUS data collection.

In a survey to validate our findings with stakeholder groups, *n* = 25 clinicians, *n* = 4 researchers and *n* = 3 policy makers responded (response rate = 61%), 87% of stakeholders indicated that they agreed to strongly agreed that the six categories provided an accurate representation of the suggested strategies to improve implementation of the FOCUS. Stakeholders also agreed that an appropriate label and description was given to each category (90 and 97% selected agree to strongly agree, respectively), 97% agreed with the prioritized list of 14 strategies, and 100% agreed with the benefits associated with each of the strategies (See Additional file 2 for more detail). The level of agreement across all questions exceeded our a priori threshold of 85%, indicating that a consensus was reached amongst our stakeholders regarding our findings.

After considering the mechanism of action of the 14 prioritized implementation strategies, all but one strategy had evidence to suggest that it would resolve the associated implementation barriers (see Additional file 4 for a detail report of the mechanism of action of each strategy). The strategy “Keep the dialogue open with clinicians to see what can be improved/changed” (see priority 7 on Table 2) has elements of three behavioral change techniques – *Problem solving*, *Review behavior goals*, *Review outcome goals*. This strategy, despite being considered important and feasible by stakeholders, was removed from the final recommended list of implementation intervention strategies because there was no empirical evidence to support that it would have an impact on the barrier *beliefs about consequences*. This intervention alone (i.e., having scheduled problem solving/review of the behavior/outcomes of the behavior) has no evidence to support its effectiveness, however, it should be noted that providing clinicians with information about the social and environmental consequences, as well as outcomes of the collected FOCUS data (e.g., priority 2 on Table 2 “Share what is done at the ministry level to look at program effectiveness using the FOCUS”) has evidence to suggest that it would impact the barrier *beliefs about consequences.*

## Discussion

To effectively improve implementation, it is important to select implementation intervention strategies that are tailored to existing barriers [1, 22]. This study contributes to an emerging body of literature that demonstrates how stakeholders can be engaged in selecting and tailoring implementation intervention strategies, something that until recently, has been referred to as a “black box” because of limited reports detailing the process [34].

Our primary research objective was to illustrate how the concept mapping approach can be used to engage stakeholders to select barrier-specific implementation strategies. Three stakeholder groups (clinicians, policymakers, researchers) participated in a concept mapping approach to brainstorm and prioritize a list of 14 strategies that could improve implementation of a clinical outcome measurement tool in pediatric speech-language pathology. To understand what barriers were being addressed by the 14 selected intervention strategies, we modified the traditional concept mapping approach.

In addition to asking clinicians to brainstorm strategy statements using a specific prompt (part of concept mapping methodology), we asked clinicians to elaborate on the barriers that they thought would be addressed by each of their suggested strategies. Specifying which barrier may be resolved by each implementation strategy is crucial because it allowed us to consider how these barriers may be impacted by specific strategies [35]. Identified barriers were mapped onto domains on the TDF and clinicians’ suggested implementation strategies addressed issues within the *beliefs about consequence* and *environmental context and resources* domains, which was consistent with the most commonly reported barriers identified in our previous study (Manuscript under review).

Our second research objective was to investigate whether the implementation strategies suggested or recommended by stakeholders were evidence informed. Based on the available literature, we considered the mechanisms of action of each of the 14 strategies prioritized by stakeholders. All but one of the prioritized strategies had evidence to suggest they would have an impact on the barriers identified by stakeholders. The final list of 13 strategies will be used to develop a detailed implementation plan in the next phases of our research [2].

This study illustrated a step-by-step approach to identifying implementation strategies that were targeted (i.e., would resolve existing barriers), important and feasible to stakeholders, and evidence-informed. In this research approach, stakeholders’ perspectives, rather than theory, guided the initial brainstorming of implementation strategies. We believe this approach was particularly appropriate in the context of our study for two reasons. First, by interviewing clinicians, we engaged stakeholders and capitalized on their knowledge of the practice context, [17, 18], allowing us to develop a focused list of strategies that would be feasible in the real-world clinical setting, and palatable to clinicians (i.e. the knowledge users). Second, we found a lack of specific details included in strategies we identified in the literature, a limitation acknowledged by others [3]. For example, *develop educational materials* is a common implementation strategy, however, to adopt this strategy we would still need to engage stakeholders to design the content and format of the materials. From our interviews, clinicians suggested specific implementation strategies such as “Provide training (e.g. case studies), so clinicians can practice completing the FOCUS consistently”. We found that our interview approach was more efficient because it generated actionable implementation strategies that took into account knowledge users’ preferences and practice contexts and, importantly, these strategies were worded in a way that was familiar to our stakeholders.

Certainly, other groups of researchers have demonstrated ways to integrate both empirical evidence and stakeholder expertise in the brainstorming and tailoring phases of implementation strategies [35, 36]. However, these approaches involve engaging all stakeholders in a discussion during an in-person meeting. This was not feasible in our study as we needed to engage stakeholders from across a large geographic region (size: 1.076 million km^2^), making it cost-prohibitive to arrange for all participants to attend in-person meetings. Our study offers an example for tailoring implementation strategies that are practice- and evidence-informed when it is not feasible to have in-person stakeholder meetings.

We made other modifications to the concept mapping approach to engage stakeholders remotely. Rather than in-person focus groups, stakeholders participated in our study via telephone interviews and web-based software, methods that may have limitations. For example, since clinicians were not able to discuss and exchange ideas in a group setting, they may have generated a lists of barriers/implementation strategies that were not exhaustive. We do not however believe this to be the case. Five clinicians disclosed having informally surveyed their colleagues for strategies to improve implementation of the FOCUS tool prior to our phone interview. To some extent, we believe their discussions with peers achieved a similar result as having focus group discussions. Additionally, we reviewed our interview transcripts and confirmed that our last four interviews did not generate any new implementation strategies (i.e. our data collection reached saturation), which suggested the interviews generated a comprehensive list of implementation strategies.

A consideration for engaging stakeholders remotely was time. A substantial amount of time was needed to transcribe the interviews conducted to identify the strategy statements generated by our participants. This introduced a significant time gap between the brainstorming stage and the sorting and rating stage. As a result, we had six clinicians choose to cease participation in the study. Although we were able to recruit three additional clinicians to participate in the sorting and rating stage, we did not have representation from all 30 service regions across all the stages of our study. To avoid the need for transcription, an alternate approach would be to ask participants to submit written statements via email or web-based software. Unlike interviews, however, there would be no opportunity for the research team to interact with participants to request clarifications, or to confirm which barriers each suggestion would addresses. In this case, the research team may need to rely on theoretical knowledge to associate implementation strategies suggested by the participants to practice barriers and validate the results through a member check step (i.e., seeking feedback from stakeholders). Finally, even though we attempted to engage all stakeholders to validate the concept map and selected implementation strategies using an online survey, we were only able to solicit feedback from 61% of our stakeholder participants. This may have impacted the external validity of our results. On-site meetings may have allowed us to engage more directly with all stakeholders during this process.

Despite the above limitations, we believe the concept mapping approach remains a powerful tool for incorporating various stakeholder views into the selection of implementation strategies. Completing the concept mapping project remotely maximized our ability to engage multiple stakeholder groups from across a wide geographic region. By remotely engaging stakeholders, we were able to provide anonymity to all participants, a challenge reported in previous work that engages multiple stakeholder groups [37]. During our interviews, clinicians generated implementation suggestions that they did not believe would be implemented by the policy-makers. For example, one clinician noted “*I recognize that probably isn’t going to be the case*” after making an implementation suggestion. Reflecting on our experience, we felt strongly that an interview approach encouraged clinicians to freely brainstorm all possible ways to improve the implementation of the FOCUS, whereas focus groups may have been more limiting due to the hierarchy of power between policy-makers (the funders) and clinicians working in the public system (the employees) [38].

Our findings also inform the evolving body of literature linking behavior change techniques and TDF domains [11]. While mapping implementation strategies to TDF domains was not the major goal of the current study, we were able to use our data from knowledge-users’ perspectives (as opposed to experts’ perspectives in the current literature [11]), to confirm an association between implementation strategies and TDF domains. One future direction for this work is to compare the association between behavior change techniques and TDF domains from the perspectives of different stakeholders (e.g., knowledge-users, implementation experts, policy makers), which may build a more accurate representation of the complex mechanism linking barriers and implementation strategies. With the list of implementation intervention strategies from this study, our team will focus on planning a system-wide implementation intervention and evaluation next [1, 2]. To evaluate the impact of the implementation intervention strategies, we will monitor changes in the identified mechanism of action of these strategies. Additionally, stakeholders will be consulted to identify and prioritize outcomes. Example outcomes may include improved implementation of the FOCUS (e.g., fidelity of FOCUS use in practice), new knowledge about the impact of services (e.g., intervention effectiveness), and individual client’s outcomes (e.g., children’s communication participation skills) [39].

## Conclusions

Our study demonstrates a real-world application of the concept mapping methodology, which we used to tailor implementation strategies to specific practice barriers. Clinicians, researchers, and policy makers across a large geographic region brainstormed and prioritized 14 important and feasible strategies they believed would improve the implementation of an outcome measurement tool in pediatric speech-language pathology. These implementation strategies were reported to resolve barriers within the *environmental context and resources* and *beliefs about consequences* domains of the Theoretical Domains Framework. Based on the best-available empirical evidence, 13 of the 14 strategies were judged to potentially have an impact on current practice barriers and were recommended for further implementation planning.

## Supplementary information

**Additional file 1.** Good Reporting of A Mixed Methods Study Checklist

**Additional file 2.** Survey to validate findings (clinician version)

**Additional file 3.** Statements included in each concept map category.

**Additional file 4.** Prioritized implementation strategies, associated barriers and mechanisms of action.

## Data Availability

De-identified datasets used and/or analysed during the current study are available from the corresponding author on reasonable request.

## Abbreviations

TDF: Theoretical Domains Framework
PSL Program: Ontario Preschool Speech and Language program
FOCUS: Focus on the Outcomes of Communication Under Six
