# Supplementary material for: Selecting and tailoring implementation interventions: a concept mapping approach
Source: BMC Health Serv Res. 2020 May 6;20:385. doi: 10.1186/s12913-020-05270-x (PMC7203846; doi:10.1186/s12913-020-05270-x)
Supplement: Supplementary file 1 — Additional file 1. Good Reporting of A Mixed Methods Study Checklist [file 12913_2020_5270_MOESM1_ESM.docx]

Additional file 1. Good Reporting of A Mixed Methods Study Checklist

| **Guideline** | **Section: page** |
| --- | --- |
| Describe the justification for using a mixed-methods approach to the research question | Background: p.6 |
| Describe the design in terms of the purpose, priority and sequence of methods | Background: p.6  Methods: p.9-14 |
| Describe each method in terms of sampling, data collection and analysis | Methods: p.9-14 |
| Describe where integration has occurred, how it has occurred and who has participated in it | Methods: p.9-14 |
| Describe any limitation of one method associated with the present of the other method | Discussion: p.23-25 |
| Describe any insights gained from mixing or integrating methods | Discussion: p.25-26 |

**Reference:** O'Cathain A, Murphy E, Nicholl J. The quality of mixed methods studies in health services research*.* J Health Serv Res Policy. 2008;13(2):92-98.
