# Supplementary material for: Selecting and tailoring implementation interventions: a concept mapping approach
Source: BMC Health Serv Res. 2020 May 6;20:385. doi: 10.1186/s12913-020-05270-x (PMC7203846; doi:10.1186/s12913-020-05270-x)
Supplement: Supplementary file 2 — Additional file 2. Survey to validate findings (clinician version) [file 12913_2020_5270_MOESM2_ESM.docx]

Additional file 2- Survey to validate findings (clinician version)

Thank you for participating in our telephone interview in late 2018 and for sharing your views and ideas about the FOCUS and how to improve its implementation. During our interview, we asked you to brainstorm ideas to improve both the collection and submission of data. As a group of 37 SLPs you generated 90 different suggestions. After the interview, you also helped us sort those 90 suggestions into categories. Based on the way you and other SLPs sorted the suggestions, we identified 6 different categories to summarize and describe SLPs’ suggestions. After the suggestions were sorted, we asked you (the SLPs) to rate both the importance and feasibility of the suggestions that were given. Members of the FOCUS research team, and Ministry representatives were also asked to rate the feasibility of the suggestions from their perspectives. As a final step in our research process, we are requesting your input one last time. First, we would like you to review the categories we identified and tell us whether they accurately represent your ideas for improving implementation of the FOCUS in the PSL Program. Second, we would like to review the suggestions that were rated as important by SLPs and as feasible by SLPs, the FOCUS research team and the Ministry and tell us whether you agree with the way they are prioritized.  This should take approximately 10-15 minutes to complete. 
Thank you for sharing your expertise with us!

- I agree to participate (1)

**Step 1:**Below you will see a summary of the categories we identified based on the 90 suggestions to improve implementation of the FOCUS given by SLPs. Category titles, definitions, and select examples of suggestions are presented.

**Category 1: Resources**-

*Definition*- providing additional financial and personnel support

*Example suggestions -*(i) hire more SLPs; (ii) provide more funding for clerical support

**Category 2: Communication**-

*Definition*- share information with frontline staff and maintain an ongoing communication between the Program and SLPs

*Example suggestions -*(i) share what is done at the ministry level to look at program effectiveness using the FOCUS; (ii) share information on how other agencies/clinicians are using FOCUS data clinically

**Category 3: FOCUS administration fidelity**-

*Definition*- improve the consistency with which the FOCUS is introduced to parents, scored, interpreted and used to support clinical practice

*Example suggestions -*(i) create a poster/visual display that explains purpose of FOCUS; (ii) make sure FOCUS scores can support functional/clinically-related activities

**Category 4: FOCUS administration logistics**-

*Definition*- facilitate the process of FOCUS data collection, as well as modify the administrative schedule of the FOCUS

*Example suggestions -*(i) offer an electronic fillable FOCUS form (e.g. on tablet/iPad/online/laptop); (ii) re-examine the frequency and timing at which FOCUS should be completed

**Category 5: FOCUS user-friendliness for parents**-

*Definition*- improve clarity, readability and literacy level of the FOCUS so that it is easier for parents to complete

*Example suggestions -*(i) improve readability of the FOCUS (e.g. increase the font size and bubble size, shading the items); (ii) simplify the wordings of FOCUS items so they are appropriate for parents' reading level

**Category 6: FOCUS comprehensiveness**-

*Definition*- ensure the FOCUS is applicable and appropriate for all children and families*-Example suggestions -*(i) make sure FOCUS items apply to all families; (ii) have separate section for items that are verbal communication vs other forms of communication

Click HERE (this contains a link to a document containing the concept map (i.e. Figure 2) and a full list of suggested strategies (i.e. Supplementary 2) to see the full list of statements in each category.

|  | Strongly Disagree | Disagree | Neither agree nor disagree | Agree | Strongly Agree |
| --- | --- | --- | --- | --- | --- |
| Q1. The labels represent the statements in each category |  |  |  |  |  |
| Q2. The definitions represent the statements in each category |  |  |  |  |  |
| Q3. These 6 categories are accurate categorization of the 90 suggestions made by SLPs |  |  |  |  |  |

Q4. Do you have any comments about the category labels?

________________________________________________________________

________________________________________________________________

________________________________________________________________

Q5. Do you have any comments about the definitions provided for each category?

________________________________________________________________

________________________________________________________________

________________________________________________________________

Q6. Do you have any comments about the categories?

________________________________________________________________

________________________________________________________________

________________________________________________________________

**Step 2**: After your interview, we asked you to rate how important and feasible the 90 suggestions offered by SLPs were. For the most part, the things that were rated as important by SLPs were also rated as feasible (e.g., SLPs thought improving the user-friendliness of the FOCUS was both important and feasible). In order to ensure the approaches to improving implementation of the FOUCS are effective, it was also important to ensure that things that were perceived as being important and feasible by SLPs were also perceived as being feasible from the perspectives of the FOCUS research team and the Ministry. Members of the FOCUS research team and the Ministry were therefore also asked to rate the feasibility of SLPs’ suggestions. Some of the suggestions that were important and feasible by SLPs were also rated as feasible by representatives from the research team and Ministry, but some were seen as not being feasible either by the Ministry or the FOCUS research team.  We have identified a list of 14 suggestions that were rated as highly important by SLPs, and feasible from the perspectives of all stakeholders. We have also prioritized the list of suggestions beginning with the one rated highest on importance and feasibility (see Table below). Next to each suggestion, we provide a numerical value of importance (averaging the rating by SLPs) and feasibility (average ratings from the FOCUS research team and the Ministry). These ratings range from 0 (not important/feasible at all) to 5 (extremely important or feasible).


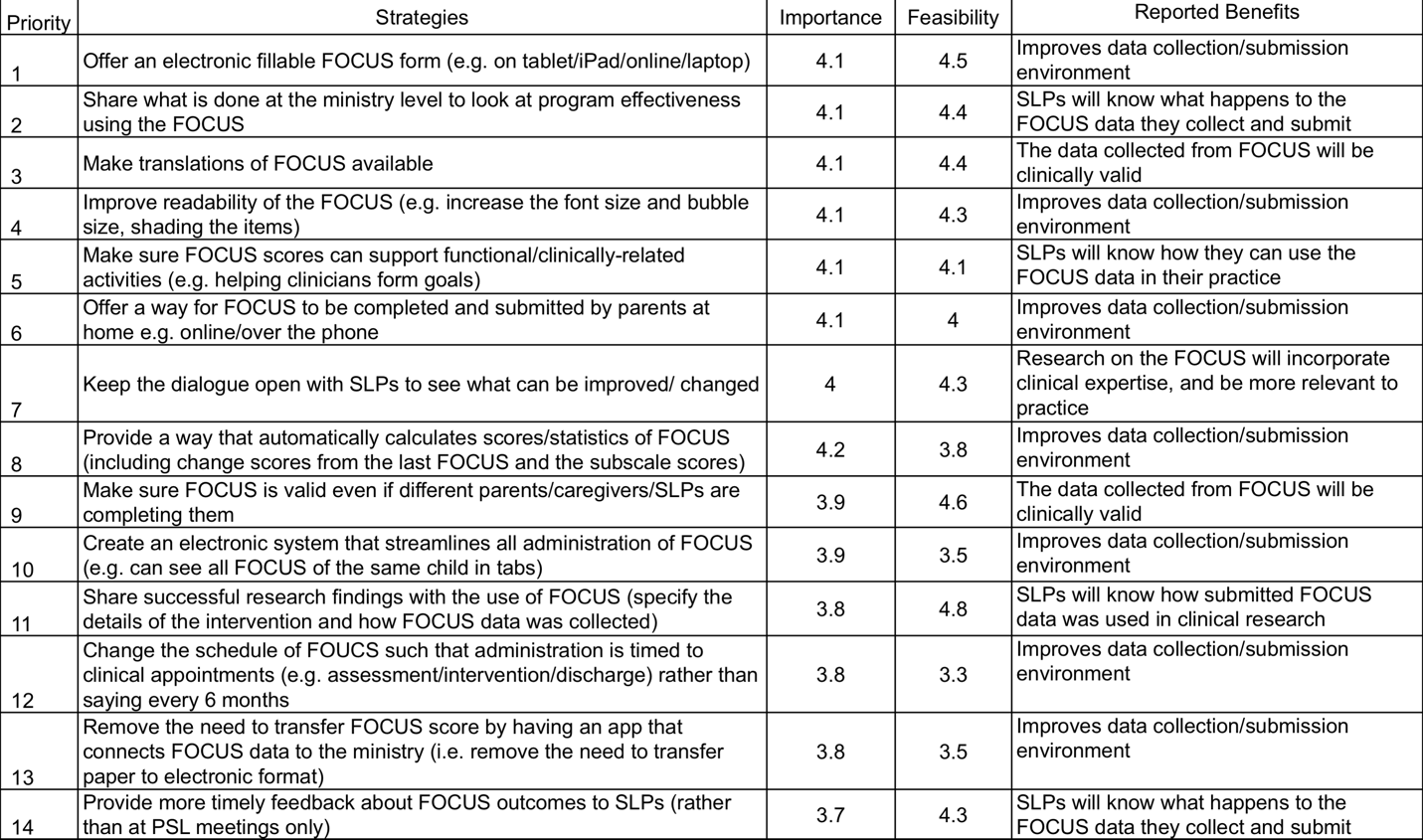


Do you agree...

|  | Strongly disagree | Somewhat disagree | Neither agree nor disagree | Somewhat agree | Strongly agree |
| --- | --- | --- | --- | --- | --- |
| Q7. with the prioritization? |  |  |  |  |  |
| Q8. with the benefits of each suggestion? |  |  |  |  |  |

Note: if the respondent selected “Strongly disagree” or “Somewhat disagree” to the prioritization, they will be asked to prioritize the list of 14 strategies.

Q9. Do you have comments about the benefits of each suggestion?

________________________________________________________________

________________________________________________________________

________________________________________________________________

**Results from survey**

*n* = 25 clinicians, *n* = 4 researchers and *n* = 3 representatives from PSL program

|  | Q1. Category labels | Q2. Category definitions | Q3.  Categories | Q7.  Prioritization | Q8.  Benefits |
| --- | --- | --- | --- | --- | --- |
| Strongly Agree | 30% | 33% | 33% | 37% | 57% |
| Agree | 60% | 63% | 53% | 60% | 43% |
| Neither agree nor disagree | 7% | 0% | 10% | 0% | 0% |
| Disagree | 0% | 0% | 0% | 3% | 0% |
| Strongly Disagree | 3% | 3% | 3% | 0% | 0% |

Participants made few comments to the open-ended questions. Seven participants made comments to Question 4 (regarding the category label chosen). Their responses are summarized below. However, since the level of agreement amongst respondents has exceeded our *a priori* threshold of consensus (i.e. 85%), we did not make changes to the category labels.

Summary of written response to Question 4:

- *n* = 4 suggested alternative labels to Category 6: FOCUS Comprehensiveness. Suggested labels include: FOCUS applicability/ appropriateness/ inclusiveness for all clients/families
- *n* = 2 recommended using one word only as category labels
- *n* = 1 recommended using “caregivers” instead of “parents” in category 5 label.
